# Magnetic resonance imaging diagnosis of leiomyomatosis peritonealis disseminata: Case report and literature review

**DOI:** 10.1016/j.radcr.2025.07.027

**Published:** 2025-08-13

**Authors:** Shanshan Li, Mingxiang Wu, Ge Dang

**Affiliations:** a Department of Radiology, Shenzhen People's Hospital, Shenzhen, China; b Department of Neurology, Shenzhen People's Hospital, Shenzhen, China

**Keywords:** Magnetic resonance imaging, Peritoneal tumor, Leiomyomatosis, Case report

## Abstract

In this study, we evaluated the magnetic resonance imaging (MRI) findings in a 44-year-old woman with leiomyomatosis peritoneal disseminata (LPD), who presented with a progressively enlarging lower abdominal mass, persistent distension, and intermittent pain several months after subtotal hysterectomy for multiple uterine tracts. MRI revealed multiple lesions below the pancreatic head, in the lower abdomen, and on abdominopelvic organ surface. The most significant lesion was located in the lower abdomen, with poorly defined boundaries relative to the anterior abdominal wall, suggesting close proximity to the peritoneum. The lesion showed heterogenous MRI signals, raising suspicion of malignancy. The diagnosis was supported by imaging features, late childbearing age, and prior leiomyoma surgery. These findings show that MRI provides distinct imaging characteristics that are valuable for LPD diagnosis, which can be validated by correlating radiological features with clinical history. This emphasizes the importance of the diagnosing LPD history and integrating imaging and patient background information to accurately identify this rare condition.

## Introduction

Leiomyomatosis peritonealis disseminata (LPD) is a rare idiopathic, hormone-dependent, benign disorder. LPD can be classified as either primary or secondary. Primary LPD has an unknown etiology, whereas secondary LPD typically develops after laparoscopic myomectomy or hysterectomy. Although LPD primarily affects women of childbearing, it does not affect women of all ages. Although LPD primarily affects women of childbearing age, a few cases have been documented in postmenopausal women, men, and young children [1,2]. The condition most commonly involves the parametrium and is frequently associated with concurrent uterine or ovarian pathologies, such as uterine The condition most commonly involves the parametrium and is frequently associated with concurrent uterine or ovarian pathologies, such as uterine leiomyomas, endometriosis, and borderline ovarian tumors [3,4]. LPD imaging findings typically reveal multiple solid or mixed cystic-solid masses of varying sizes distributed across the peritoneum, mesentery, greater omentum, and ovarian tumors [4]. mesentery, greater omentum, rectouterine pouch, and abdominopelvic organ surfaces. The magnetic resonance imaging (MRI) signal characteristics of LPD resemble those of uterine organs. LPD resemble those of uterine leiomyomas, with evidence of degeneration, hemorrhage, cystic changes, and mixed signals, indicating potential malignant transformation. malignant transformation.

The exact pathogenesis of LPD remains unknown. Factors such as excessive estrogen secretion, leiomyoma enucleation, hysterectomy for leiomyomas, and genetic predisposition may contribute to LPD development [5,6]. Diagnostic approaches typically include imaging modalities such as ultrasound, computed tomography (CT), and MRI, which facilitate the preliminary disease identification. However, a definitive diagnosis requires histological examination [7].

We aimed to analyze the MRI findings of a patient with LPD and review the relevant literature, given the limited number of reports available on the imaging diagnosis of LPD.

## Case presentation

A 44-year-old woman who had undergone a subtotal hysterectomy 2 years earlier, was admitted to our hospital 15 d after the discovery of a space-occupying mass in her pelvic cavity. She had no history of other major illnesses and denied having chronic conditions, such as hypertension or diabetes.

Physical examination results revealed resonance of the lung percussions and clear breath sounds without moist rales. A cardiac examination revealed no abnormalities, a heart rate of 85 beats/min, normal rhythm, and no pathological murmurs. A cardiac examination revealed no abnormalities, a heart rate of 85 beats/min, normal rhythm, and no pathological murmurs. A longitudinal surgical scar approximately 13 cm in length and laparoscopic puncture marks with slight local bulging were observed in the lower A hard abdomen. A longitudinal surgical scar approximately 13 cm in length and laparoscopic puncture marks with slight local bulging were observed in the lower abdomen. A hard, hypomobile, 10 × 10 cm, solid mass without tenderness was palpable in the right lower abdomen. A hard, hypomobile, 10 × 10 cm, solid mass without tenderness was palpable in the right lower abdomen. The liver and spleen were not palpable below the costal margin, and no tenderness was noted. The bowel sounds and renal area examination results were within normal limits.

Laboratory findings indicated a slightly elevated carbohydrate antigen 125 (CA-125) level of 47.00 U/mL (normal range: 0-30.2 U/mL).

MRI results revealed postoperative changes in the uterus and multiple nodules with abnormal signal intensities distributed below the pancreatic head, in the lower abdomen, and within the pelvic cavity. The largest lesion was located on the right side of the pelvis and it measured approximately 101 × 71 mm. The lesion displayed T1 hypo-intensity, T2 heterogeneous hypo-intensity, and patch-like long T2 signals with clear boundaries. Restricted diffusion was observed. Contrast-enhanced findings indicated prominent heterogeneous enhancement. The lesion was relatively demarcated from the uterus but poorly demarcated from the aneurysm. The lesion was relatively demarcated from the uterus but poorly demarcated from the anterior parietal peritoneum. An irregular cystic lesion, measuring approximately 62 × 40 mm, was found posteroinferior to the mass. The results of contrast-enhanced scanning showed no enhancement of the cystic shadow, although 2 ring-shaped enhancing foci, with a larger Restricted diffusion was not observed. A large mixed-signal shadow (63 × 55 mm) showed T1 A large mixed-signal shadow (63 × 55 mm) showed T1 heterogeneous hypo-intensity, T2 heterogeneous hyper-intensity, and ring-shaped enhancement. The lesion was well-demarcated from the uterus and rectum on contrast-enhanced imaging. Multiple nodular cystic cavities without enhancing foci were observed in the cervix and vagina.

MRI revealed multiple leiomyoma-like masses on the surfaces of the abdominopelvic organs. The patient was diagnosed with LPD, considering her history of leiomyoma surgery and status of late childbearing age. The patient was diagnosed with LPD, considering her history of leiomyoma surgery and status of late childbearing age.

The histological analysis of the tumor revealed abundant smooth muscle cells with mild atypia and mitotic activity (up to 8 per 10 high-power fields), along with infarct formation. The histological analysis of the tumor revealed abundant smooth muscle cells with mild atypia and mitotic activity (up to 8 per 10 high-power fields, along with infarct formation. Immunohistochemical analysis revealed positive staining for smooth muscle actin, desmin, Ki-67 (30%), P16 (40%), and P53 (scattered cells) and negative staining for CD10, CD117, melan-A, and HMB45.

No tumors were detected in the left internal iliac lymph nodes, although chronic inflammation was identified in the residual cervix. Additionally, chronic inflammation of the fallopian tubes and formation of ovarian inclusion cysts, consistent with hydrosalpinx changes and corpus albicans. Additionally, chronic inflammation of the fallopian tubes and formation of ovarian inclusion cysts, consistent with hydrosalpinx changes and corpus albicans formation, were observed. The results of the study were summarized in the following table.

**Fig. 1:**
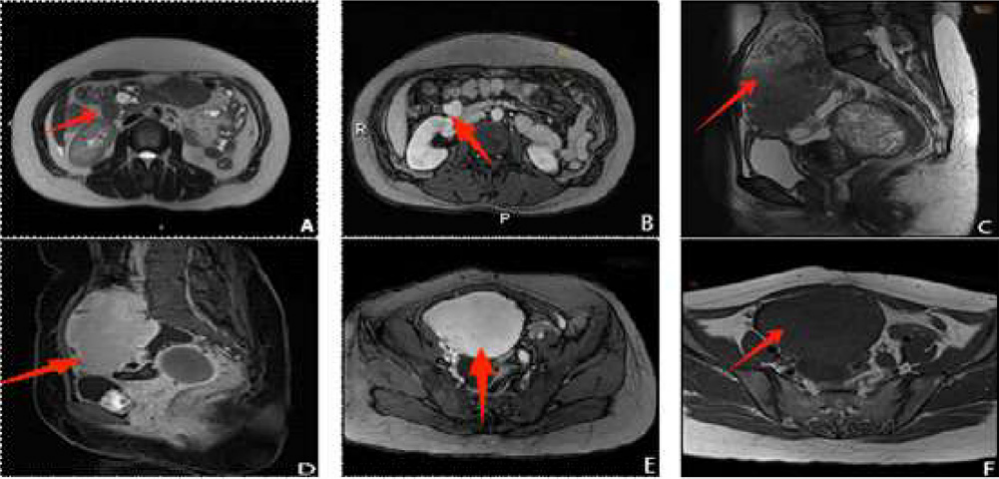
Magnetic resonance imaging (MRI) findings of a 44-year-old woman with a history of subtotal hysterectomy for multiple uterine leiomyomas 2 years The findings of a 44-year-old woman with a history of subtotal hysterectomy. A. Axial T2-weighted imaging (T2WI) demonstrating a hypo-intense nodule located below the pancreatic head (arrow). B. Contrast-enhanced delayed-phase axial MRI showing prominent homogeneous enhancement of the nodule (arrow), with smooth margins and enhancement similar to the myometrium. C. Sagittal T2WI revealing a large mass in the lower abdomen and anterior pelvic cavity (arrow) exhibiting heterogeneous signal intensity. D. Contrast-enhanced venous-phase sagittal MRI illustrating significant enhancement of the solid portion of the lesion, comparable to that of the myometrium, and highlighting a close relationship between the lesion and the anterior parietal peritoneum (arrow). myometrium, and highlighting a close relationship between the lesion and the anterior parietal peritoneum (arrow). E. Contrast-enhanced venous-phase transverse MRI indicating prominent homogeneous enhancement of the pelvic lesion (arrow), with a nonenhancing cystic focus posterior to the lesion. F. Transverse T1WI depicting the mass as hypo-intense with smooth, sharply defined edges (arrow).

The patient underwent abdominopelvic tumor resection under general anesthesia after a comprehensive examination. The intraoperative findings included contracture and nodules in the greater omentum, tumor implantation in the mesentery and other areas, solid masses in the right pelvic cavity and rectouterine pouch adhesions at the residual tissues, adhesions at the lateral and lateral surfaces of the abdomen. The intraoperative findings included contracture and nodules in the greater omentum, tumor implantation in the mesentery and other areas, solid masses in the right pelvic cavity and rectouterine pouch adherent to the surrounding tissues, adhesions at the residual cervix with the absence of the uterine body, and a normal appearance of All the surgical procedures were completed without complications. Histological examination confirmed a leiomyoma. Certain regions exhibited increased tumor cell density and mitotic figures; however, no necrosis was observed (Fig. 1).

## Discussion

LPD is a rare, benign, and proliferative disease characterized by the formation of numerous solid lesions in the smooth muscle and connective tissue. These lesions are widely distributed along the peritoneum of the abdominopelvic cavity [8]. LPD was first described by Wilson and Peale in 1952 and later named by Taubert et al. in 1965. To date, more than 200 such cases have been documented [9].

The histogenesis and etiology of LPD remain unclear and may be associated with increased levels of sex hormones. The histological manifestations of LPD are diverse and are typically characterized by multiple smooth muscle cell hyperplasia on the peritoneal surface, with a mild cellular morphology and clear borders, possibly accompanied by collagen fiber deposition or edema. The pathology of the patient in this study exhibited a large number of smooth muscle cells with mild atypia, mitotic figures, and infarcted foci. This suggested high proliferative activity and a potential risk of malignant transformation. Given the patient’s history of laparoscopic myomectomy, this finding was consistent with iatrogenic LPD. Although the histology showed active mitotic figures, no extensive necrosis or vascular invasion was observed. The final pathological diagnosis remained leiomyoma, suggesting that malignant transformation should be excluded by multiparametric evaluation.

LPD lesions typically exhibit multiple or diffuse distribution patterns, with sizes ranging from a few millimeters to several centimeters [10]. LPD frequently involves the pelvic peritoneum, particularly the rectouterine pouch, and may affect other peritoneal sites, such as the mesentery, greater omentum, and retroperitoneal space. Unlike malignant peritoneal tumors, ascites are usually absent or minimal in patients with LPD [11]. The MRI findings in patients with LPD exhibit specific characteristics. These typically include T1 iso-intensity or hypo-intensity, T2 hypo-intensity or slight hyper-intensity, and T2 hyper-intensity. intensity or slight hyper-intensity, and signal heterogeneity, particularly in the presence of hemorrhage, necrosis, or other malignant features [12]. Peripheral hyper-intensities or iso-intensities may also be observed on T2-weighted imaging (T2WI).

In the present case, MRI revealed multiple abnormal signal shadows below the pancreatic head, in the lower abdomen, and in the pelvic cavity. These findings suggested the peritoneal dissemination of leiomyomas and the presence of multiple lesions. findings suggested the peritoneal dissemination of leiomyomas and the presence of multiple lesions. The largest lesion, in the right pelvic cavity, showed plain and heterogeneous enhancement on contrast-enhanced although. The largest lesion, in the right pelvic cavity, showed plain and heterogeneous enhancement on contrast-enhanced imaging. Although the lesion was clearly demarcated from the uterus, heterogeneous enhancement indicated an uneven vascular distribution. Although the lesion was clearly demarcated from the uterus, heterogeneous enhancement indicated an uneven vascular distribution and necrotic areas within the mass. These findings suggested the possibility of malignant transformation.

An irregular cystic shadow was observed posteroinferior to the largest lesion. Although the cystic shadow showed no enhancement on contrast imaging, 2 ring-shaped enhancing foci were noted within the cyst, suggesting the presence of hemorrhagic or necrotic areas surrounded by proliferating tissue. Although the cystic shadow showed no enhancement on contrast imaging, 2 ring-shaped enhancing foci were noted within the cyst, suggesting the presence of hemorrhagic or necrotic areas surrounded by proliferating granulation tissue. In addition, a large mixed-signal shadow in the rectouterine space displayed T1 heterogeneous hypo-intensity and T2 heterogeneous hyper-intensity. Contrast-enhanced imaging revealed a ring-shaped enhancement with a relatively clear boundary, indicating that the Contrast-enhanced imaging revealed a ring-shaped enhancement with a relatively clear boundary, indicating that the leiomyoma had invaded the rectouterine region without causing extensive disruption of the adjacent structures. In the present case, the patient had a history of laparoscopic leiomyoma resection. The patient presented with multiple masses in the abdominopelvic cavity upon admission. examination revealed abundant tumor cells with mild atypia, mitotic activity and infarct formation. These findings, combined with the clinical These findings, combined with the clinical history of the patient, led to a diagnosis of iatrogenic LPD. The possibility of malignant transformation could not be ruled out [13,14]. In the present case, the MRI signal characteristics closely overlapped with those typically observed in leiomyomas, with the largest mass exhibiting The diagnosis of LPD was confirmed through integration of the MRI findings, pathological results, laboratory data, and surgical history [14].

LPD must be differentiated from other conditions, including ovarian cancer, gastrointestinal malignancies, peritoneal metastases, multiple primary sarcomas of the abdominal cavity, and gastrointestinal mesenchymal tumors. Although LPD often involves the ovaries, LPD typically affects only the ovarian surface and does not invade the parental cavity. Although LPD often involves the ovaries, LPD typically affects only the ovarian surface and does not invade the parenchyma. Consequently, the MRI of patients with LPD demonstrates the ovaries and follicles, with masses exhibiting well-defined boundaries, and the ovarian surface is not a part of the parenchyma. masses exhibiting well-defined boundaries, smooth and sharp margins, and relatively preserved surrounding fat spaces [15]. In contrast, ovarian cancer often appears as a solid or cystic mass on MRI, with mixed and heterogeneous internal signals, indistinct boundaries, and poor separation from adjacent tissues. Advanced ovarian cancer frequently invades surrounding structures, such as the fallopian tubes, uterus, and small intestine, and is commonly accompanied by a large number of other organs. Advanced ovarian cancer frequently invades surrounding structures, such as the fallopian tubes, uterus, and small intestine, and is commonly accompanied by ascites. In gastrointestinal malignancies, peritoneal lesions typically spare the muscularis propria, and malignant tumors often exhibit hypo-intensity on T1WI and hyper-intensity on T2WI. The standard features include irregular morphology, blurred margins, and invasion. The standard features include irregular morphology, blurred margins, and invasion of the surrounding tissues. The results of contrast-enhanced imaging may reveal heterogeneous enhancement that sharply contrasts the surrounding normal tissues. The results of contrast-enhanced imaging may reveal heterogeneous enhancement that sharply contrasts the surrounding normal tissues. LPD generally involves both muscular and peritoneal lesions, although rare cases may present as isolated abdominal masses. Such cases must be distinguished from multiple primary sarcomas of the abdominal cavity, including liposarcomas, leiomyosarcomas, and malignant fibrous histiocytes. Such cases must be distinguished from multiple primary sarcomas of the abdominal cavity, including liposarcomas, leiomyosarcomas, and malignant fibrous histiocytomas [16]. Malignant transformation occurs in approximately 2%-5% of people with LPD, and differentiating its imaging characteristics from those of Malignant transformation occurs in approximately 2%-5% of people with LPD, and differentiating its imaging characteristics from those of primary abdominal sarcomas is challenging when necrosis or marked heterogeneous enhancement is observed on contrast-enhanced scans. Peritoneal metastases with distinct boundaries often manifest as multiple nodular, mass-like, or ``dirt-like'' changes in the greater Peritoneal metastases with distinct boundaries often manifest as multiple nodular, mass-like, or ``dirt-like'' changes in the greater omentum and mesentery. Their MRI features typically include slow enhancement, which is often moderate to pronounced in the delayed phases. peritoneal metastases generally appear hyper-intense or iso-intense on fat-suppressed T2WI, distinguishing these from the characteristic MRI findings of LPD [17]. findings of LPD [17]. Gastrointestinal mesenchymal tumors, primarily originating in the gastrointestinal tract, may involve the omentum or mesenteric membrane. These tumors are usually solitary, prone, and have been shown to be associated with a variety of other diseases. These tumors are usually solitary, prone to cystic necrosis, and appear as T2 mixed-hyper-intense lesions with smooth boundaries. Contrast-enhanced imaging frequently shows a peripheral enhancement pattern that enables its differentiation from LPD. Contrast-enhanced imaging frequently shows a peripheral enhancement pattern that enables its differentiation from LPD.

In summary, LPD should be considered when MRI findings reveal multiple leiomyoma-like tumors distributed on the surfaces of abdominopelvic organs, particularly in patients with a history of laparoscopic myomectomy or uterine surgery. The absence of systemic symptoms such as weight loss, ascites, or liver metastasis further supports this diagnosis. The absence of systemic symptoms such as weight loss, ascites, or liver metastasis further supports this diagnosis. facilitate the preoperative diagnosis of LPD and enable effective treatment planning.

## Author contributions

Guarantor of the integrity of the entire study: Shanshan Li. Study concept and design: Shanshan Li. Literature research: Mingxiang Wu. clinical studies: Shanshan Li and Ge Dang. Experimental studies/data analyses: Shanshan Li and Mingxing Wu. Statistical analysis: N/A. Manuscript preparation: Shanshan Li. Manuscript editing: Shanshan Li.

## Statement of funding

Not applicable.

## Ethical declaration

Informed consent was obtained from the patient; This study has been approved by the hospital's ethics committee, and the informed consent of all participants has been obtained. The full name of the ethics committee I am studying and the research institute it belongs to are: Ethics committee of Shenzhen People's Hospital.

## Clinical trial registration number in cases of clinical trials

Not applicable.

## STS number in case of student IJMR

Not applicable.

## Data sharing statement for all original research

All the original data can be shared.

## Declaration of artificial intelligence (AI) in scientific writing

The author declares that no artificial intelligence was used for writing assistance in this case report.

## Patient consent

The patients involved in the article have given their informed consent.
